# Zinc- and fluoride-containing bioactive glass enhances angiogenesis-mediated bone regeneration via M2d macrophage activation

**DOI:** 10.1038/s41598-026-44931-5

**Published:** 2026-04-13

**Authors:** Koki Otake, Takeru Kondo, Hiroaki Kakinuma, Yumi Sato, Sara Ambo, Amal Ashry, Kulapatch Engkatanachai, Jun Sato, Hiroshi Egusa

**Affiliations:** 1 Division of Molecular & Regenerative Prosthodontics, Tohoku University Graduate School of Dentistry, 4-1 Seiryomachi, Aoba-Ku, Sendai-City, 980-8575 Japan; 2 Department of Next-Generation Dental Material Engineering, Tohoku University Graduate School of Dentistry, Sendai, 980-8575 Japan; 3 Center of Excellence for Dental Stem Cell Biology, Faculty of Dentistry, Chulalongkorn University, Bangkok, 10330 Thailand

**Keywords:** Bioactive glass, M2d macrophages, Angiogenesis, Bone regeneration, Zinc, Fluoride, Biotechnology, Cell biology, Diseases

## Abstract

Angiogenesis is crucial for bone regeneration because it supplies oxygen and nutrients to developing tissues. Immune cells, particularly macrophages, play an important role in regulating vascularization. The M2d macrophage subtype, which is phenotypically similar to tumor-associated macrophages, is characterized by high vascular endothelial growth factor (VEGF) expression; however, its role in regenerative angiogenesis and bone regeneration remains unclear. In this study, we developed a zinc- and fluoride-containing phosphate-based bioactive glass (ZFBG) by incorporating ZnO, CaF_2_, and Al_2_O_3_ into a phosphate glass network. Compared with conventional silicate-based bioactive glass (BG45S5), ZFBG exhibited superior ion release and solubility under physiological conditions. In vitro, ZFBG polarized macrophages toward the VEGF-producing M2d phenotype, and conditioned media from these macrophages significantly enhanced endothelial cell migration. In a mouse calvarial defect model, ZFBG increased M2d macrophage accumulation, CD31^+^ neovascularization, bone volume, and bone mineral density relative to BG45S5. ZFBG showed minimal direct effects on human umbilical vein endothelial cells, suggesting that its proangiogenic effects are primarily macrophage mediated. Collectively, these findings suggest that ZFBG is associated with enhanced angiogenesis-related bone regeneration, potentially through modulation of VEGF-producing macrophages, and highlight the promise of ion-releasing bioactive glasses for immune-mediated bone repair.

**Supplementary Information:**

The online version contains supplementary material available at 10.1038/s41598-026-44931-5.

## Introduction

Bone regeneration relies on both osteogenesis and a functional vascular network to deliver nutrients, oxygen, and signaling molecules^1^. Angiogenesis is a critical determinant of successful bone healing, particularly during the early inflammatory and reparative phases^2^. Although it was previously believed that osteoblasts coordinate the coupling between bone formation and neovascularization^3^, recent studies indicate that immune cells, particularly macrophages, are key regulators of bone vascularization^4^.

Macrophages are heterogeneous and exhibit plasticity in response to microenvironmental cues^5^. Broadly, they can be classified as pro-inflammatory M1 or anti-inflammatory/tissue-regenerative M2 types^6^. In bone regeneration, macrophages regulate inflammation and repair processes^7^. Among the M2 subsets, M2d macrophages share phenotypic and functional similarities with tumor-associated macrophages (TAMs), notably producing vascular endothelial growth factor (VEGF) to promote angiogenesis^8^. M2d macrophages promote angiogenesis in the context of tumor progression^9^. Under pathological conditions, such as cancer, M2d macrophages facilitate tumor progression by promoting vascularization^10^. M2d macrophages also aid in wound healing and tissue repair by supporting neovascularization and re-epithelialization^11^. Despite their well-established role in pathological angiogenesis, their significance in physiological bone healing remains unclear and merits further investigation.

Bone tissue engineering employs various biomaterials to support bone regeneration, often by targeting immune responses^12^. In particular, bioactive glasses release biologically active ions to modulate cellular behavior^13^. Bioactive glass 45S5 (BG45S5) is a silicate-based glass that is widely used to promote osteogenesis^14^. However, its limited degradation properties and low ion release under physiological conditions have restricted clinical applications^15^. To address this, a new phosphate-based bioactive glass was developed with higher aqueous solubility and enhanced ion release compared with conventional silicon dioxide (SiO₂)-based glasses. Specifically, this glass is formulated using zinc oxide (ZnO) and calcium fluoride (CaF₂) to enhance the delivery of zinc and fluoride ions. Additionally, aluminum oxide (Al₂O₃) is incorporated to enable precise control over solubility and ion-release characteristics. We previously reported that this zinc- and fluoride-releasing phosphate-based bioactive glass (ZFBG) promotes osteoblast differentiation and modulates macrophage responses, enhancing bone regeneration^16^.

Zinc ions regulate angiogenesis by modulating immune cell behavior, such as enhancing VEGF production in monocytes, which supports angiogenesis and bone regeneration^17^. Meanwhile, fluoride ions influence macrophage polarization, promoting the M2 phenotype to create a favorable anti-inflammatory microenvironment for bone regeneration^18^. Hence, ZFBG may regulate VEGF expression by polarizing macrophages toward the M2d phenotype, potentially functioning as an immunomodulatory material that promotes angiogenesis through VEGF signaling. However, whether ZFBG specifically induces M2d macrophages to drive angiogenesis remains unclear, and the role of M2d macrophages in bone regeneration via immune-driven neovascularization requires further analysis.

In this study, we investigated the effects of ZFBG on macrophage polarization and angiogenesis using a murine calvarial defect model. We focused on M2d macrophage induction and their potential contribution to angiogenesis-mediated bone regeneration.

## Results

### Physicochemical features of ZFBG compared with BG45S5

**Fig. 1:**
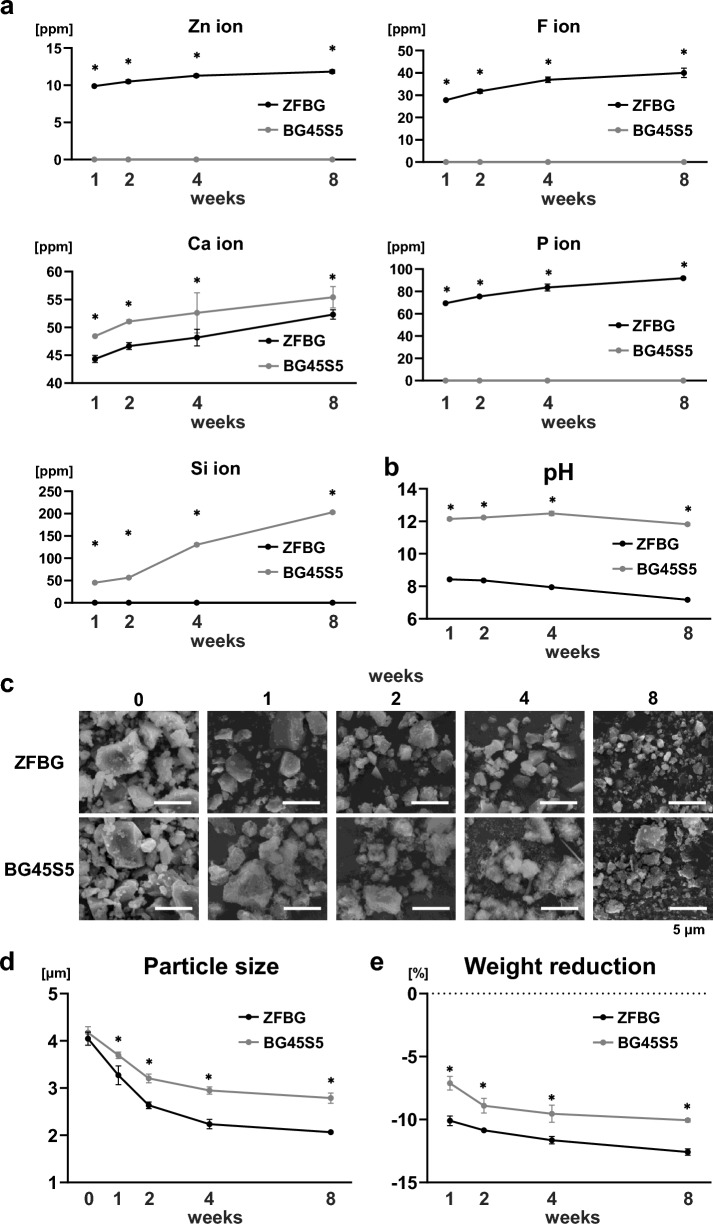
Physicochemical features of zinc- and fluoride-releasing bioactive glass (ZFBG) compared with Bioglass45S5 (BG45S5). **(a)** The concentrations of Zn, F, Ca, P, and Si ions released into the supernatant were measured after immersion of ZFBG (50 mg) or BG45S5 (50 mg) in distilled water (2 mL) (*n* = 7). **(b)** The pH of the supernatant was measured after immersion of ZFBG or BG45S5 in distilled water (*n* = 5). **(c)** Representative scanning electron microscopy (SEM) images show the reduction in particle size of ZFBG or BG45S5 in distilled water (scale bars: 5 µm). **(d)** The median particle diameter of ZFBG or BG45S5 after immersion in distilled water was quantified (*n* = 5). **(e)** The weight reduction ratio of ZFBG or BG45S5 after immersion in distilled water was also determined (*n* = 5). Data are expressed as mean ± standard deviation (SD), and statistical comparisons were performed using Student’s *t*-test at each time point. *P < 0.05 indicates a statistically significant difference.

ZFBG was fabricated by mixing and sintering KH₂PO₄, ZnO, CaF₂, Al₂O₃, and SiO₂. Elemental ion release profiles showed that ZFBG continuously released Zn, F, Ca, and P ions into distilled water over 8 weeks, whereas BG45S5 primarily released Ca and Si ions. However, BG45S5 released more Ca ions than ZFBG (Fig. 1a). When immersed in distilled water at pH 7.0, the pH of the ZFBG supernatant increased to approximately 8.0 after 1 week, whereas that of the BG45S5 supernatant rose sharply to ~ 12.0, indicating strong alkalinity (Fig. 1b). Scanning electron microscopy (SEM) revealed a more pronounced reduction in particle size for ZFBG than for BG45S5 during immersion (Fig. 1c). Quantitative analysis confirmed a significantly smaller median particle diameter for ZFBG than for BG45S5 at each time point (Fig. 1d). Furthermore, the weight loss ratio of ZFBG was significantly greater than that of BG45S5 throughout the immersion period (Fig. 1e). These findings suggest that ZFBG exhibits a sustained ion release profile and higher solubility under physiological pH conditions compared with BG45S5.

### Effects of ZFBG and BG45S5 on macrophage polarization and VEGF expression

**Fig. 2:**
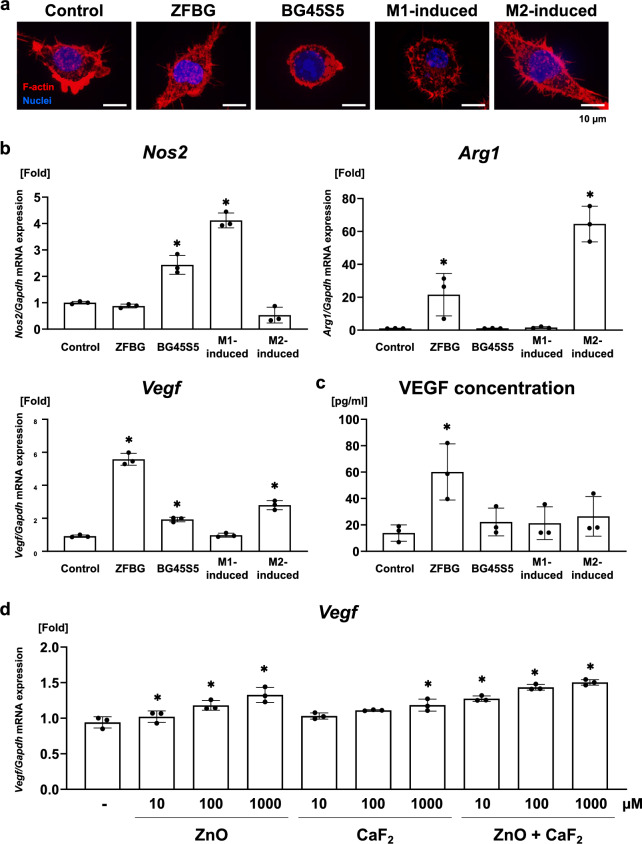
Effects of ZFBG and BG45S5 on macrophage polarization and VEGF expression. **(a)** Representative immunofluorescence images of J774A.1 cells cultured with ZFBG or BG45S5 and M1- or M2-induced cells are shown, stained for F-actin and nuclei. **(b)** Relative gene expression of *Nos2*, *Arg1*, and *Vegf*, normalized against *Gapdh*, was determined in J774A.1 cells cultured with ZFBG or BG45S5 and M1- or M2-induced cells for 1 day using reverse transcription-quantitative polymerase chain reaction (RT-qPCR) (*n* = 3). **(c)** VEGF protein concentration in the culture supernatant of J774A.1 cells and M1- or M2-induced cells after 1 day of culture with ZFBG or BG45S5 was measured using enzyme-linked immunosorbent assay (*n* = 3). **(d)** Relative *Vegf* expression normalized against *Gapdh* was measured in J774A.1 cells cultured for 1 day with ZnO, CaF₂, or ZnO + CaF_2_ using RT-qPCR (*n* = 3). Data are expressed as mean ± SD. Statistical comparisons were performed using one-way ANOVA with Tukey’s multiple comparisons test for panels (**a**–**c**), and Dunnett’s test was used to compare experimental groups with the control group (0 µM) in panel **(d)**. *P < 0.05 indicates statistical significance.

J774A.1 cells, a murine macrophage line, exhibited spindle-shaped morphology similar to that of interleukin (IL)-4-induced M2 macrophages when cultured with ZFBG, whereas cells cultured with BG45S5 had rounded morphology resembling that of lipopolysaccharide (LPS)/interferon-gamma (IFN-γ)-induced M1 macrophages (Fig. 2a, Supplementary Fig. S1). Importantly, no significant reduction in cell viability was observed in either the ZFBG- or BG45S5-treated groups compared with the control group (Supplementary Fig. S2), indicating that the observed phenotypic changes were not attributable to acute cytotoxic effects. The differences in cell morphology between the ZFBG- and BG45S5-treated groups were consistent with their gene expression profiles. Specifically, J774A.1 cells treated with ZFBG showed significantly higher expression of *Arg1*, a marker of M2 macrophages, than the control group. In contrast, BG45S5-treated cells exhibited significantly elevated expression of *Nos2*, a marker of M1 macrophages (Fig. 2b). These findings suggest that ZFBG promotes M2 polarization of macrophages, whereas BG45S5 favors M1 polarization. Furthermore, ZFBG significantly upregulated *Vegf* mRNA expression and VEGF protein secretion, whereas BG45S5 induced a modest increase in *Vegf* expression (Fig. 2b), without a corresponding increase in protein secretion (Fig. 2c). Thus, ZFBG facilitates macrophage polarization toward a VEGF-producing M2 phenotype, known as M2d macrophages.

J774A.1 cells were cultured with ZnO, CaF₂, or a combination of both to elucidate the role of individual ions released from ZFBG. Both ZnO and CaF_2_ enhanced *Vegf* expression in a dose-dependent manner, with ZnO showing a stronger effect than CaF_2_. Notably, the combination of ZnO and CaF₂ augmented *Vegf* expression compared with either ion alone (Fig. 2d).

### Effects of ZFBG and BG45S5 on human umbilical vein endothelial cell (HUVEC) migration

**Fig. 3:**
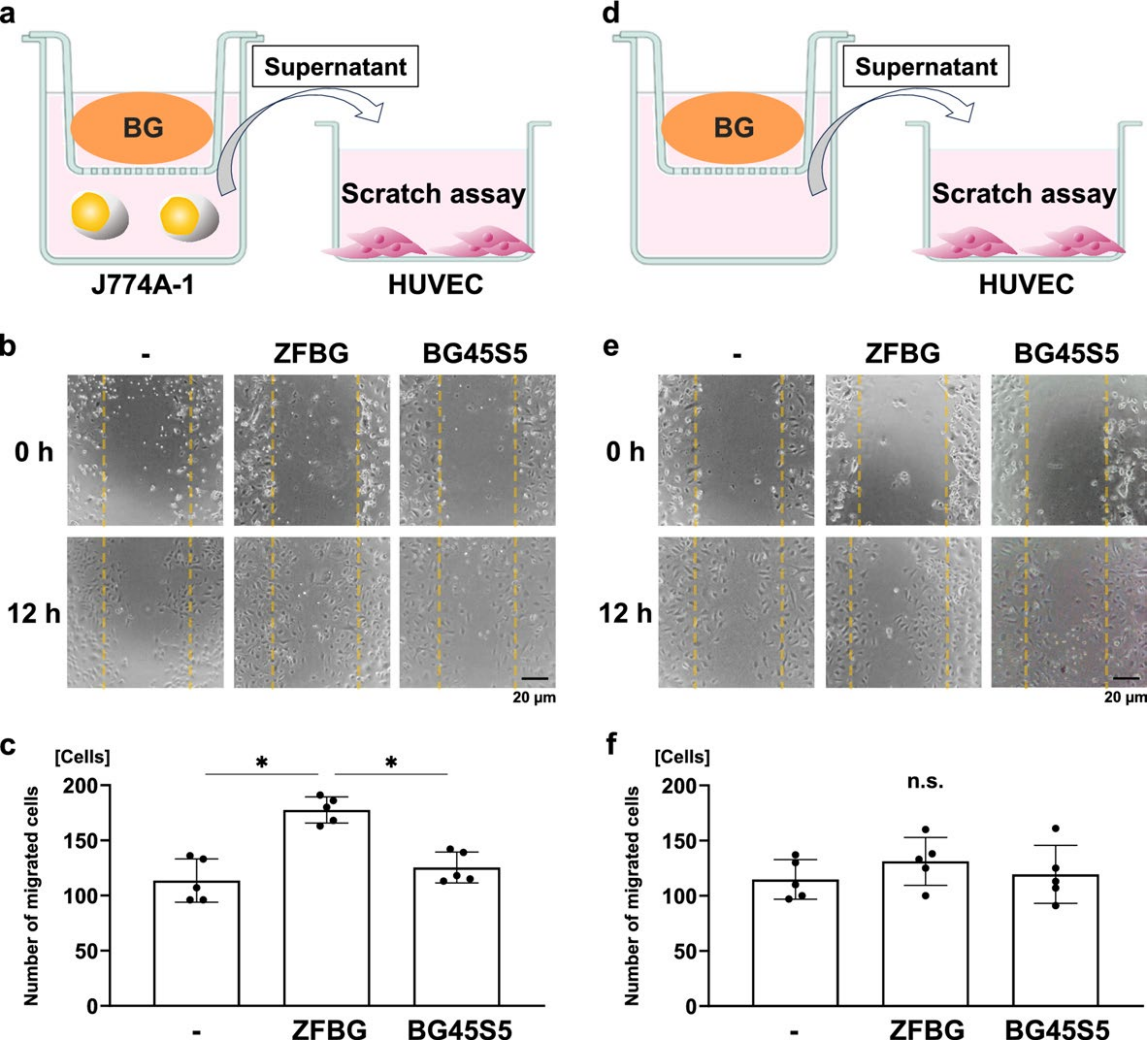
Effects of ZFBG and BG45S5 on the migration of human umbilical vein endothelial cells (HUVECs). **(a)** A schematic illustration shows the scratch assay using HUVECs cultured in the conditioned medium from J774A.1 cells previously treated with ZFBG or BG45S5 for 1 day. **(b)** Representative phase-contrast images of HUVEC migration at 0 and 12 h after scratching are shown, with yellow dotted lines indicating the scratched areas (scale bars: 20 µm). **(c)** Quantification of HUVECs migrating into the scratched area at 12 h is shown (*n* = 5). **(d)** A separate schematic illustrates the scratch assay using HUVECs cultured in dissolution medium obtained from ZFBG or BG45S5. **(e)** Representative phase-contrast images of HUVEC migration at 0 and 12 h in medium containing dissolved ions from ZFBG or BG45S5 are shown (scale bars: 20 µm), and** (f)** migration was quantified at 12 h (*n* = 5). Data are expressed as mean ± standard deviation (SD). Statistical comparisons were performed using one-way ANOVA with Tukey’s multiple comparisons test. *P < 0.05 indicates statistical significance.

A scratch assay was performed using HUVECs cultured in conditioned medium collected from J774A.1 cells previously treated with ZFBG or BG45S5 to assess the effect of ZFBG on endothelial cell migration mediated by macrophages (Fig. 3a). HUVECs exposed to the conditioned medium from ZFBG-treated macrophages exhibited significantly enhanced migration compared with the control group, whereas the BG45S5 group showed no such enhancement (Fig. 3b, c).

A separate scratch assay was performed using HUVECs cultured in the dissolution medium obtained from ZFBG or BG45S5 in the absence of macrophages to evaluate the direct effect of bioactive glasses on HUVEC migration (Fig. 3d). Neither ZFBG nor BG45S5 dissolution medium promoted HUVEC migration compared with the control (Fig. 3e, f). Thus, ZFBG indirectly enhanced HUVEC migration via soluble factors secreted by macrophages, most likely VEGF, rather than directly stimulating endothelial cells.

### Effects of ZFBG and BG45S5 on bone regeneration and angiogenesis in a mouse calvarial defect model

**Fig. 4:**
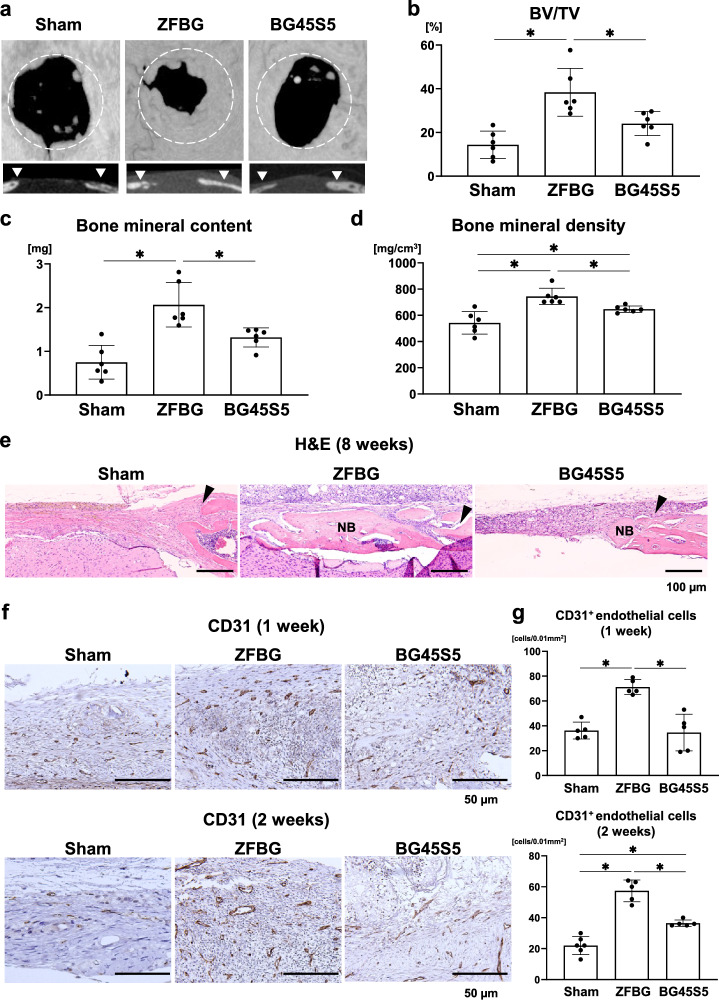
Effects of ZFBG and BG45S5 on bone regeneration and angiogenesis in a mouse calvarial defect model. **(a)** Representative 3-dimensional micro-computed tomography (micro-CT) images (upper panels) and corresponding frontal sections (lower panels) of calvarial defects at 8 weeks post-operation are shown, with white dotted lines indicating the defect areas and arrowheads denoting the defect margins. **(b–d)** Quantitative analysis of bone volume/tissue volume (BV/TV) (**b**), bone mineral content **(c)**, and bone mineral density **(d)** within the defect area at 8 weeks post-operation is shown (*n* = 5). **(e)** Representative hematoxylin and eosin (H&E) staining images of calvarial defects at 8 weeks are shown (scale bars: 200 µm), with arrowheads indicating defect margins. **(f)** Representative immunohistochemical staining images for CD31 in calvarial defects at 1 and 2 weeks post-operation are shown (scale bars: 50 µm), and **(g)** CD31-positive endothelial cells within a 0.01 mm^2^ area were quantified at 1 and 2 weeks (*n* = 5). Data are expressed as mean ± standard deviation (SD). Statistical comparisons were performed using one-way ANOVA with Tukey’s multiple comparisons test. *P < 0.05 indicates statistical significance.

ZFBG or BG45S5 was implanted into critical-sized calvarial bone defects in mice to evaluate the in vivo bone regenerative capacity of the bioactive glasses. Micro-computed tomography (micro-CT) imaging revealed that ZFBG implantation led to greater mineralized tissue formation than BG45S5 at 8 weeks post-surgery (Fig. 4a). Quantitative analysis confirmed that the ZFBG group exhibited significantly higher BV/TV (Fig. 4b), bone mineral density (Fig. 4c), and bone mineral content (Fig. 4d) than the BG45S5 group. Histological analysis supported micro-CT findings. Substantial new bone formation was observed in the ZFBG-implanted defects, whereas only minimal bone regeneration was found in the BG45S5 group (Fig. 4e, Supplementary Fig. S3).

Immunohistochemical staining for CD31 was performed to assess the angiogenic potential of these materials during bone healing. At 1 and 2 weeks post-surgery, the number of CD31-positive endothelial cells was significantly higher in the ZFBG group than in the BG45S5 and control groups (Fig. 4f, g, Supplementary Fig. S4). Thus, ZFBG promoted bone regeneration in part by enhancing angiogenesis during the early phase of healing.

### Effects of ZFBG and BG45S5 on M2d macrophages in a mouse calvarial defect model

**Fig. 5:**
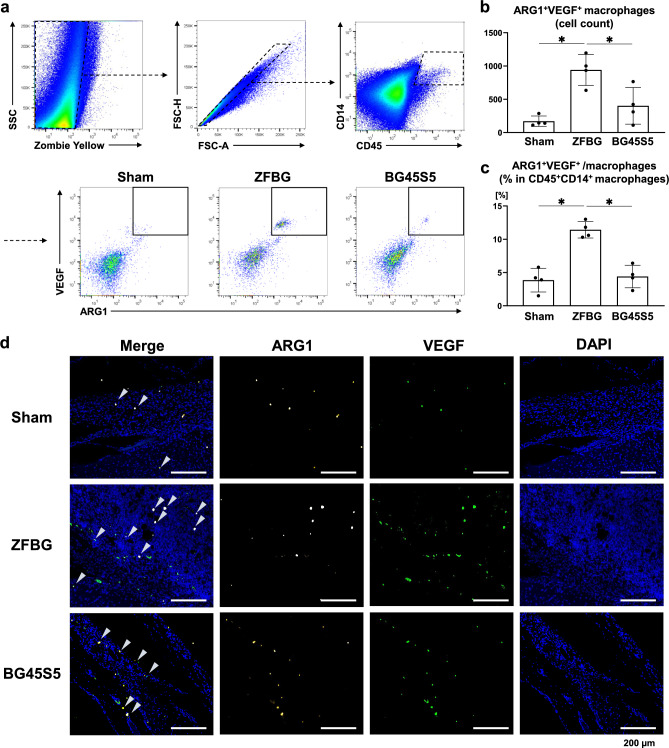
Effects of ZFBG and BG45S5 on M2d macrophages in a mouse calvarial defect model. **(a)** The gating strategy for flow cytometric analysis of M2d macrophages in calvarial defects is shown, with M2d macrophages defined as CD45^+^CD14^+^ARG1^+^VEGF^+^ cells. **(b)** The absolute number of M2d macrophages in calvarial defects is shown (*n* = 4), as well as the **(c)** proportion of M2d macrophages among CD45⁺CD14⁺ macrophages (*n* = 4). **(d)** Representative immunofluorescence images of calvarial defects at 1 week post-operation are shown, stained for ARG1 (yellow), VEGF (green), and nuclei (DAPI) (scale bars: 200 µm). Data are expressed as mean ± standard deviation (SD). Statistical comparisons were performed using one-way ANOVA with Tukey’s multiple comparisons test. *P < 0.05 indicates statistical significance.

Flow cytometry and immunofluorescence analyses were performed using tissues harvested from calvarial defects at 1 week post-implantation. M2d macrophages were defined as CD45^+^CD14^+^ARG1^+^VEGF^+^ cells based on an established gating strategy (Fig. 5a). Flow cytometric analysis revealed that the number of M2d macrophages was significantly higher in the ZFBG group than in the BG45S5 and control groups (Fig. 5b). Moreover, the proportion of M2d macrophages among total CD45^+^CD14^+^ macrophages increased significantly in the ZFBG group (Fig. 5c). Immunofluorescence staining supported these findings. Numerous ARG1^+^VEGF^+^ double-positive M2d macrophages were observed within the defect area in the ZFBG group, whereas only a few such cells were detected in the BG45S5 and control groups (Fig. 5d, Supplementary Fig. S5). Thus, ZFBG promoted in vivo polarization of macrophages toward a VEGF-producing M2d phenotype, which may have contributed to enhanced angiogenesis and bone regeneration.

## Discussion

In this study, we demonstrated that a novel ZFBG enhanced angiogenesis-mediated bone regeneration by promoting angiogenesis via immunomodulation. ZFBG drives macrophage polarization toward the VEGF-producing M2d subtype, leading to increased endothelial cell migration and accelerated bone regeneration in a murine calvarial defect model. These findings highlight the role of M2d macrophages as key mediators of immune regulation and neovascularization during bone regeneration.

The M2d macrophage subtype is characterized by its proangiogenic capacity through VEGF production^19^. Although M2d macrophages are well studied in the context of cancer^20^, their function in physiological or regenerative processes remains unclear. The present study shows that bioactive material-induced M2d macrophages improve bone healing by enhancing angiogenesis. ZFBG implantation increased ARG1⁺VEGF⁺ M2d macrophages and CD31⁺ endothelial cells in bone defects, resulting in greater new bone formation. Thus, M2d macrophages, under non-pathological conditions, may serve as therapeutic targets for bone regeneration.

M2d macrophages aid tissue repair in multiple organs, not just bone. These TAM-like cells promote repair through VEGF secretion, improving vascularization in skin wounds^21^ and fostering mucosal epithelial tissue recovery in ulcerative colitis by expressing VEGF and IL-10^22^. They may also contribute to muscle regeneration^23^, consistently supporting angiogenesis across diverse tissues. The consistency of their function highlights the translational potential of targeting M2d polarization in biomaterial-based therapies.

Although VEGF is a well-established proangiogenic mediator secreted by M2d macrophages, it likely does not account for all angiogenic effects observed in this study. TAMs, which share phenotypic and functional similarities with M2d macrophages, produce several soluble factors, including matrix metalloproteinase-9 (MMP-9), hypoxia-inducible factor (HIF-1α), tumor necrosis factor (TNF)α, transforming growth factor (TGF)β, adrenomedullin, and basic fibroblast growth factor. These mediators contribute to vascular sprouting, endothelial cell survival, and vessel maturation^24^. For instance, MMP-9 is pivotal in remodeling the extracellular matrix^25,26^, and HIF-1α enhances endothelial cell activation and migration in hypoxic microenvironments, facilitating neovascularization^27^. Meanwhile, TNFα^28^, TGF-β^29^, adrenomedullin^30^, and basic fibroblast growth factor^31^ promote robust neovascularization, particularly in tumor microenvironments. Therefore, ZFBG-induced M2d macrophages likely secrete additional angiogenic molecules beyond VEGF. Future studies using proteomic profiling of macrophage-conditioned media and loss-of-function assays targeting multiple angiogenic pathways are needed to identify the full spectrum of ZFBG-induced immunoangiogenic signals and support the development of bioactive materials targeting the immunovascular axis.

ZFBG activated M2d macrophages through the release of zinc, fluoride, and calcium. Emerging evidence supports the critical role of these ions in regulating M2d macrophages. Zinc deficiency in the tumor microenvironment impairs TAM phagocytic activity and VEGF secretion, whereas supplementation restores their proangiogenic phenotype^32,33^. Similarly, fluoride ions promote M2-like polarization and VEGF production, indicating a potential role in driving M2d-like responses^34^. Intracellular calcium-binding proteins are involved in TAM-mediated angiogenesis by regulating VEGF signaling pathways^35^. These findings provide mechanistic support for the observed upregulation of M2d macrophages and VEGF secretion in ZFBG-treated groups, emphasizing the importance of ion-mediated immune regulation in biomaterial-guided tissue regeneration.

Although previous studies have demonstrated that zinc stimulates angiogenesis in HUVECs^36^, the proangiogenic effects of ZFBG were not observed in the current study in direct stimulation assays. This discrepancy may be attributed to differences in zinc concentrations. A previous study reported that 60 µM zinc stimulates HUVECs^37^, whereas approximately 150 µM (10 ppm) zinc was released by ZFBG in vitro, which may have inhibited endothelial cell function. This supports the biphasic effect of zinc in endothelial cells: lower concentrations (< 80 µM) promote proliferation and migration, whereas higher concentrations (> 100 µM) attenuate these functions^38^. Endothelial activation was mediated by M2d macrophages, highlighting their critical intermediary role in the cellular cascade from bioactive glass dissolution to angiogenesis. This further highlights the importance of considering immune responses, beyond osteoblast activity, in the design and evaluation of biomaterials for bone regeneration.

Despite the promising findings, this study has certain limitations that warrant consideration. First, the use of a single macrophage cell line (J774A.1) may not fully recapitulate the diversity of macrophage responses in vivo. Future studies employing primary macrophages and other macrophage cell lines will be essential to validate the observed immunoangiogenic effects. Moreover, although ZFBG-induced M2d polarization was associated with enhanced angiogenesis and bone regeneration, the intracellular signaling pathways underlying this polarization remain to be elucidated. Loss-of-function approaches, including VEGF inhibition and M2d-targeted strategies, as well as genetically modified or macrophage-depletion mouse models, will be necessary to directly establish the causal role of M2d macrophages in vivo. In addition, ZFBG may influence not only M2d macrophages but also other immune cell populations that collectively contribute to tissue regeneration. Therefore, comprehensive analyses, such as single-cell RNA sequencing of defect tissues, will be required to clarify the broader immunological landscape and the coordinated cellular responses induced by ZFBG.

In conclusion, this study suggests a potential role for M2d macrophages in bone regeneration and supports the immunoangiogenic potential of ZFBG. Our results indicate that ZFBG is associated with enhanced early neovascularization and subsequent bone formation, possibly through modulation of the immune microenvironment toward a VEGF-producing macrophage phenotype. These findings may inform the development of next-generation bioactive materials that leverage immune–vascular coupling to achieve efficient and predictable bone regeneration.

## Methods

### Preparation of ZFBG

ZFBG was synthesized using potassium dihydrogen phosphate (KH_2_PO_4_), ZnO, CaF_2_, Al_2_O_3_, and SiO_2_ (all from FUJIFILM Wako Pure Chemical Corporation, Osaka, Japan). The ZFBG composition comprised 27% P_2_O_5_, 13% CaO, 8% ZnO, 26% F, 4% Al_2_O_3_, and 22% K_2_O (Supplementary Table S1). The raw materials were weighed into a platinum crucible and melted at 1100 °C for 60 min in an electric furnace (MF-1, GC, Tokyo, Japan). The molten glass was poured onto a steel plate and compressed with a steel press. After slow cooling, the glass was crushed in a ball mill (zirconia beads, 5 mm diameter) until the particles were approximately 4 µm.

### Ion release and pH evaluation

Polyether cell culture inserts (Corning, NY, USA) containing 50 mg of synthesized ZFBG or BG45S5 (G018-144, SCHOTT, Landshut, Germany) were placed in 12-well culture plates with 2 mL ion-exchanged water (pH 7.0) per well and incubated at 37 °C. Ion concentrations (Na, K, Ca, Al, Si, P, Zn) in the supernatants were measured via inductively coupled plasma optical emission spectrometry (ICP-OES, iCAP-6300, Thermo Fisher Scientific, Waltham, MA, USA). Fluoride ions were quantified using a fluoride ion electrode (LAQUA F-72, HORIBA, Kyoto, Japan), and pH was measured using a pH meter (LAQUA F-72, HORIBA).

### Evaluation of the weight reduction ratio (WR)

Glass suspensions were aspirated onto filter paper (GC-50, ADVANTEC, Tokyo, Japan) and dried at 130 °C for 1 h. The weight reduction ratio (WR) was calculated using Eq. (1):

1 $${\text{WR }}\left( \% \right){\text{ }} = {\text{ }}\left( {{\text{1 }}{-}{\text{ WB}}/{\mathrm{WA}}} \right){\text{ }} \times {\text{ 1}}00$$

where WA is the initial weight before immersion and WB is the final dry weight after immersion.

### Particle size analysis

Dried glass powders were suspended in 100% ethanol and sonicated for 15 min using an ultrasonic bath (US-105; SND, Nagano, Japan). Particle size distribution was measured with a laser diffraction particle size analyzer (Mastersizer 3000, Malvern Panalytical, Malvern, UK) under continuous stirring at 500 rpm.

### Scanning electron microscopy

Dried glass powders were sputter-coated with platinum and observed via SEM (JSM-6390LA, JEOL, Tokyo, Japan) at an accelerating voltage of 10 kV.

### Macrophage polarization assay

Murine macrophage J774A.1 cells (JCRB9108; Japanese Collection of Research Bioresources Cell Bank, Tokyo, Japan) were cultured in Dulbecco’s Modified Eagle’s Medium (DMEM; Nacalai Tesque, Kyoto, Japan) supplemented with 10% heat-inactivated fetal bovine serum (HFBS; Thermo Fisher Scientific), 2 mM L-glutamine, 100 U/mL penicillin, and 100 μg/mL streptomycin (all from FUJIFILM Wako Pure Chemical Corporation) at 37 ℃ with 5% CO_2_. Cells were seeded at 2 × 10^5^ cells/mL in 12-well plates and incubated for 24 h. Polyether cell culture inserts containing ZFBG (50 mg) or BG45S5 (50 mg) were added and incubated for another 24 h. M1 polarization was induced with 100 ng/mL LPS from *Escherichia coli* (Sigma-Aldrich, St. Louis, MO, USA) and 10 ng/mL recombinant mouse IFN-γ (FUJIFILM Wako Pure Chemical Corporation) for 24 h (M1 control); M2 polarization was achieved with 20 ng/mL recombinant mouse interleukin (IL)-4 (FUJIFILM Wako Pure Chemical Corporation) for 24 h (M2 control). Total RNA was extracted for qRT-PCR using TaqMan probes (primer details in Supplementary Table S1).

For immunofluorescence, cells were seeded at 5 × 10^4^ cells/mL in 50 mm glass-bottom dishes (D911600, Matsunami Glass Ind., Osaka, Japan), treated with ZFBG (50 mg) or BG45S5 (50 mg), fixed in 10% neutral-buffered formalin (FUJIFILM Wako Pure Chemical Corporation), stained with rhodamine phalloidin (1:400) and Hoechst 33,258 (1:500, Thermo Fisher Scientific), and imaged by confocal microscopy (LSM 780, Carl Zeiss, Jena, Germany).

### Enzyme-linked immunosorbent assay (ELISA) for VEGF quantification

After incubation with ZFBG (50 mg) or BG45S5 (50 mg) for 24 h, the culture supernatants were collected. VEGF levels were quantified using an ELISA kit (KE10009, Proteintech, Rosemont, IL, USA) following the manufacturer’s protocol.

### Evaluation of the effects of ions on VEGF expression

J774A.1 cells were cultured and stimulated using polyether cell culture inserts containing ZnO, CaF₂, or a combination of both. After 24 h, RNA was extracted, and qRT-PCR was performed.

### Endothelial cell migration assay

HUVECs (American Type Culture Collection [ATCC], Manassas, VA, USA) were cultured in DMEM with 10% HFBS, 50 μg/mL heparin, 50 μg/mL endothelial cell growth supplement (FUJIFILM Wako Pure Chemical Corporation), 100 U/mL penicillin, and 100 μg/mL streptomycin. Cells (1.4 × 10^5^ cells/mL) were scratched with a 200 μL pipette tip, washed, and cultured for 12 h in macrophage-conditioned medium or dissolution medium obtained from ZFBG (50 mg) or BG45S5 (50 mg) cultures. Migrated HUVECs in the scratched area were counted.

### Animal care

The Tohoku University Animal Research Committee (Approval No. 2022DnA-008–04) approved all animal experiments. All animals had free access to regular rodent diet and water and were maintained in standard housing conditions in accordance with the relevant guidelines and regulations. Mice were humanely euthanized by cervical dislocation to harvest their calvariae. All animal experiments were improved in accordance with the updated ARRIVE 2.0 guidelines.

### Mouse calvarial defect model and micro-CT analysis

Bilateral 3 mm circular calvarial defects were created in 8-week-old C57BL/6 J mice (CLEA Japan, Tokyo, Japan) using a trephine bar (Dentach, Tokyo, Japan) under isoflurane anesthesia (FUJIFILM Wako Pure Chemical Corporation). ZFBG (2 mg) or BG45S5 (2 mg) was implanted into the bone defects with gelatin gel (Cellmatrix, Nitta Gelatin, Osaka, Japan). Sham controls received gelatin alone. After 8 weeks, calvariae were harvested for micro-CT analysis (ScanXmate-E090, Comscan, Kanagawa, Japan) at 70 kVp and 100 mA with a 1-mm-thick brass filter. Bone volume, bone mineral content, and bone mineral density were quantified using TRI/3D-BON software (Ratoc System Engineering, Tokyo, Japan).

### Histological analyses of regenerated bone in calvarial defects

Decalcified calvarial bone samples were paraffin-embedded, sectioned (5 μm), and stained with hematoxylin (Muto Pure Chemicals, Tokyo, Japan) and eosin (Muto Pure Chemicals). For CD31 immunostaining, antigen retrieval was performed in Tris–EDTA (pH 9.0). Sections were treated with anti-CD31 (1:2000, EPR17259, Abcam, Cambridge, UK), followed by HRP-conjugated secondary antibody (Santa Cruz Biotechnology, Dallas, TX, USA) and 3,3´-diaminobenzidine (Thermo Fisher Scientific).

For immunofluorescence analysis, sections were stained with anti-VEGF (1:50, AAM51, R&D Systems, Minneapolis, MN, USA) and anti-arginase 1 (ARG1; 1:100, D4E3M, CST, Danvers, MA, USA), followed by Alexa Fluor® 488 and 555 secondary antibodies (Abcam). Nuclei were counterstained with 4′,6-diamidino-2-phenylindole (DAPI; DOJINDO, Kumamoto, Japan) and visualized via fluorescence microscopy (LSM 780, Carl Zeiss).

### Flow cytometry

Calvariae (1 × 1.5 mm, including defects) were minced and digested with 1 mg/mL collagenase II (Worthington Biochemical, Lakewood, NJ, USA), 0.1 mg/mL DNase I (Roche Diagnostics GmbH, Mannheim, Germany), and 10 mg/mL bovine serum albumin (Sigma-Aldrich). After 20 min at 37 °C, the digested tissue was filtered through 70 µm and 40 µm strainers. Red blood cells and dead cells were removed using ACK Lysing Buffer (Thermo Fisher Scientific) and a dead cell removal kit (Miltenyi Biotec, North Rhine-Westphalia, Germany), respectively. Nonspecific binding was blocked with anti-mouse CD16/32 (93, BioLegend, San Diego, CA, USA). Cells were stained with Zombie Yellow™ (BioLegend), anti-mouse CD45 (30-F11, BioLegend), and anti-mouse CD14 (Sa14-2, BioLegend).

For intracellular staining, cells were treated with a Cell Activation Cocktail containing Brefeldin A (BioLegend), permeabilized with the Cutoff-Fast™ Fix/Perm Buffer Set (BioLegend), and stained with anti-ARG1 (A7R34, eBioscience) and anti-VEGF (AAM51, R&D Systems). Flow cytometry was performed using a BD LSR Fortessa, and data were acquired using FACS Diva (Becton Dickinson, Franklin Lakes, NJ, USA). Raw data were analyzed using FlowJo (Becton Dickinson).

### Statistical analyses

Data were expressed as mean ± standard deviation (SD). Statistical significance was assessed using Student’s *t*-test or one-way analysis of variance (ANOVA) with Tukey’s or Dunnett’s post-hoc test. The Shapiro–Wilk test was used to assess normal distribution. A *P* value < 0.05 was considered statistically significant. Sample sizes are described in the figure legends.

## Supplementary Information

Supplementary Information 1.

Supplementary Information 2.

Supplementary Information 3.

Supplementary Information 4.

## Data Availability

All data generated or analysed during this study are included in this published article and its Supplementary information file.
