# Supplementary material for: Zinc- and fluoride-containing bioactive glass enhances angiogenesis-mediated bone regeneration via M2d macrophage activation
Source: Sci Rep. 2026 Apr 13;16:11351. doi: 10.1038/s41598-026-44931-5 (PMC13077007; doi:10.1038/s41598-026-44931-5)
Supplement: Supplementary file 2 — Supplementary Information 2. [file 41598_2026_44931_MOESM2_ESM.docx]

**Supplementary Table S1: Primers used for TaqMan-based PCR**

| Gene | Accession number |
| --- | --- |
| *Arg1* | Mm00475988_m1 |
| *Nos2* | Mm00440502_m1 |
| *Vegfa* | Mm00437306_m1 |
| *Gapdh* | Mm99999915_g1 |
