# Supplementary material for: Zinc- and fluoride-containing bioactive glass enhances angiogenesis-mediated bone regeneration via M2d macrophage activation
Source: Sci Rep. 2026 Apr 13;16:11351. doi: 10.1038/s41598-026-44931-5 (PMC13077007; doi:10.1038/s41598-026-44931-5)
Supplement: Supplementary file 3 — Supplementary Information 3. [file 41598_2026_44931_MOESM3_ESM.docx]

**Supplementary Figures and Legends**

**Supplementary Figure S1.** Low magnification immunofluorescence images of J774A.1 cells cultured with ZFBG or BG45S5 and M1- or M2-induced cells are shown, stained for F-actin and nuclei.

**Supplementary Figure S2.** WST-1-based evaluation of cell number of J774A.1 cells cultured in the with ZFBG or BG45S5 (*n* = 3).

**Supplementary Figure S3.** Low magnification H&E staining images of calvarial defects at 8 weeks are shown (scale bars: 200 µm), with arrowheads indicating defect margins.

**Supplementary Figure S4.** Low magnification immunohistochemical staining images for CD31 in calvarial defects at 1 and 2 weeks post-operation are shown (scale bars: 50 µm).

**Supplementary Figure S5.** High magnification immunofluorescence images of calvarial defects from the ZFBG group at 1 week post-operation are shown, stained for ARG1 (yellow), VEGF (green), and nuclei (DAPI) (scale bars: 20 µm).

**Supplementary table S1: Compositions of the glasses**

|  | Multicomponent phosphate (MP) glass | Bioglass 45S5 |
| --- | --- | --- |
| P_2_O_5_ (mol%)  SiO_2_ (mol%)  CaO (mol%)  ZnO (mol%)  F (mol%)  Al_2_O_3_ (mol%)  K_2_O (mol%)  Na_2_O (mol%) | 27  -  13  8  26  4  22  - | 5  45  25  -  -  -  -  25 |
