# Supplementary material for: Zinc- and fluoride-containing bioactive glass enhances angiogenesis-mediated bone regeneration via M2d macrophage activation
Source: Sci Rep. 2026 Apr 13;16:11351. doi: 10.1038/s41598-026-44931-5 (PMC13077007; doi:10.1038/s41598-026-44931-5)
Supplement: Supplementary file 4 — Supplementary Information 4. [file 41598_2026_44931_MOESM4_ESM.pdf]

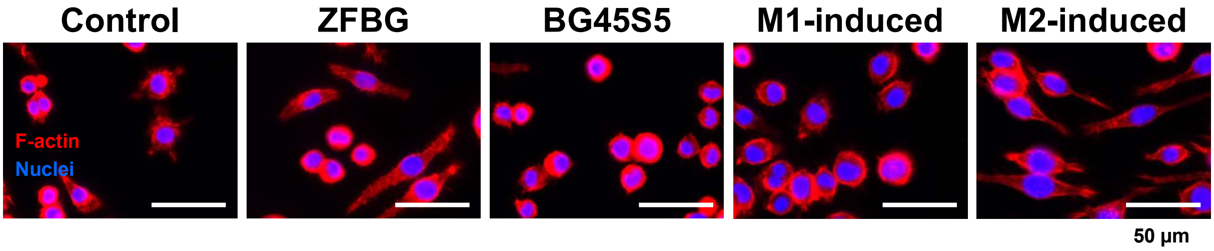

Supplementary Figure S1

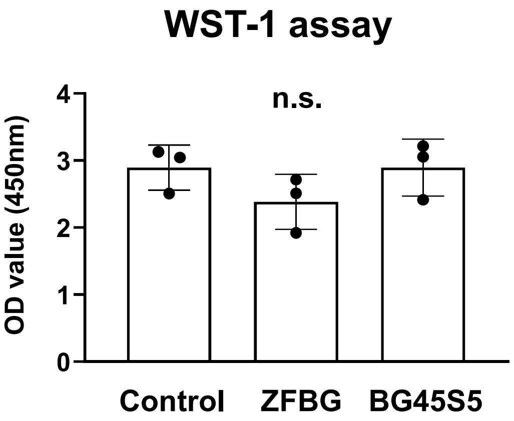

Supplementary Figure S2

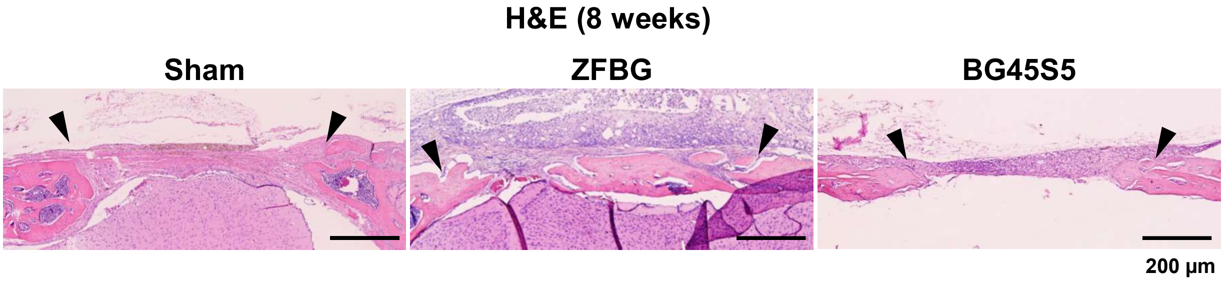

Supplementary Figure S3

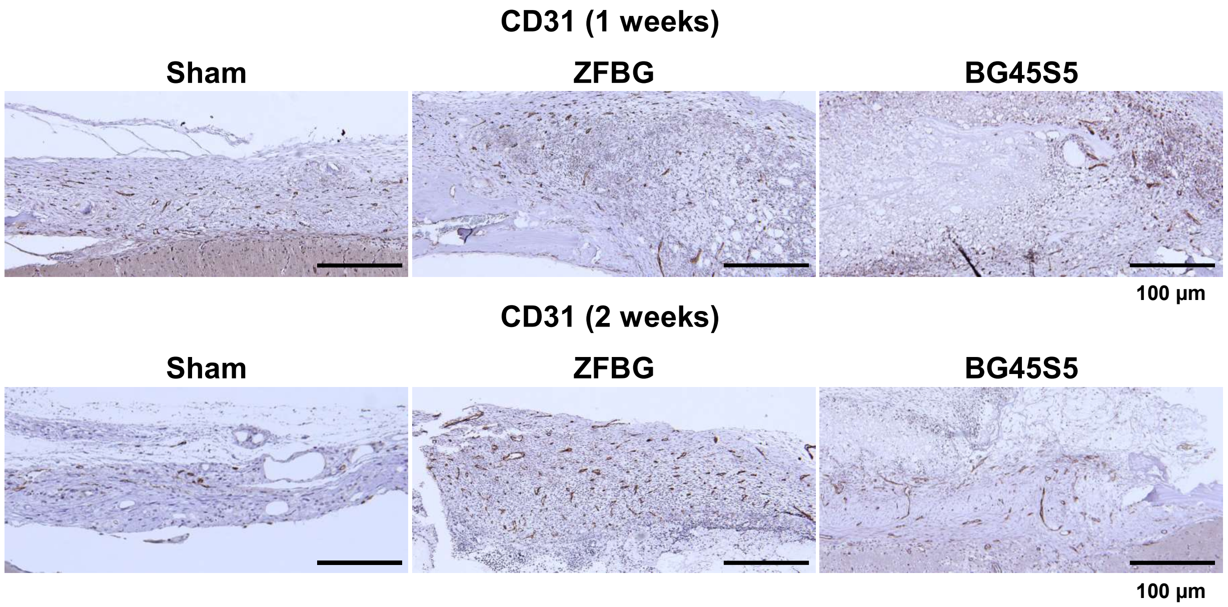

Supplementary Figure S4

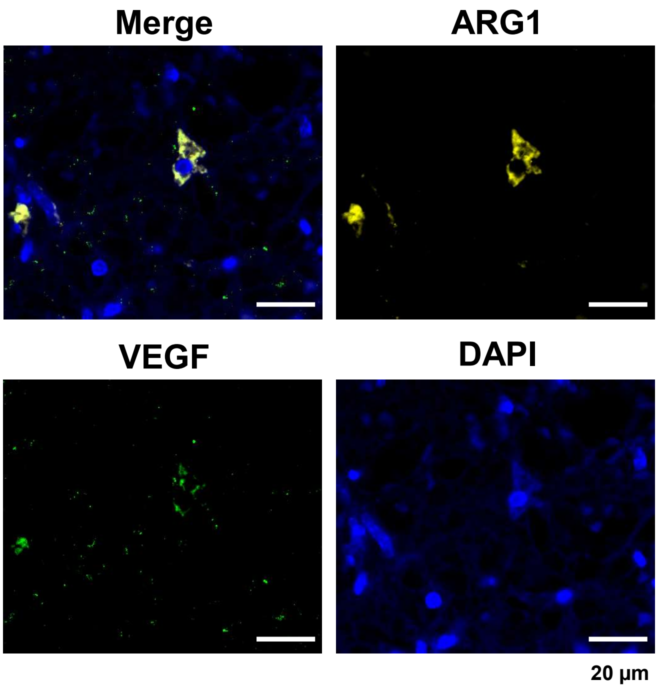

Supplementary Figure S5
